# Exploration of Digital Interventions for Vaping Cessation: Scoping Review

**DOI:** 10.2196/76983

**Published:** 2025-10-23

**Authors:** Karlee Fonteyne, Elizabeth Keys, Khalad Hasan, Laura Struik

**Affiliations:** 1 School of Nursing, Faculty of Health and Social Development, University of British Columbia, 1147 Research Road, Kelowna, BC, V1V 1V7, Canada, 1 250-807-9972; 2 Department of Computer Science, Mathematics, Physics and Statistics, Irving K. Barber Faculty of Science, University of British Columbia, Kelowna, BC, Canada

**Keywords:** vaping cessation, digital interventions, mobile health, text messaging programs, contingency management, behaviour change, youth and young adults, e-cigarette cessation, telehealth

## Abstract

**Background:**

Digital interventions have emerged as a promising approach to support vaping cessation, particularly among youth and young adults. Mobile apps, text messaging programs, telehealth-delivered contingency management, and web-based or social media interventions offer scalable and accessible alternatives to traditional cessation methods. However, there is considerable variation in how these interventions are designed, implemented, and evaluated, with inconsistencies in engagement strategies, theoretical frameworks, and long-term effectiveness.

**Objective:**

This scoping review aimed to map the current landscape of digital interventions for vaping cessation and identify key strategies, effectiveness outcomes, and implementation challenges. The following questions were addressed: (1) What digital interventions have been developed or evaluated for vaping cessation? (2) What evidence exists regarding the effectiveness of these digital interventions in promoting vaping cessation and user engagement? (3) What key barriers and facilitators influence the adoption, adherence, and success of digital vaping cessation interventions? (4) What gaps remain in the literature, and what areas should future research prioritize to enhance the design and effectiveness of digital vaping cessation tools?

**Methods:**

This scoping review followed the Joanna Briggs Institute methodology and adhered to the PRISMA-ScR (Preferred Reporting Items for Systematic Reviews and Meta-Analyses extension for Scoping Reviews) guidelines. A systematic search was conducted in CINAHL, MEDLINE, and PsycINFO using search terms related to vaping cessation and digital health interventions. Studies examining mobile apps, text messaging programs, social media or web-based interventions, and telehealth coaching explicitly designed for vaping cessation were included. A narrative synthesis was conducted to identify common themes, barriers, and facilitators.

**Results:**

Sixteen studies were identified, including SMS text messaging programs, mobile apps, telehealth-delivered contingency management, and web-based or social media interventions. Many interventions reported moderate to high abstinence rates. Programs incorporating personalized messaging, behavioral tracking, and social and interactive features demonstrated greater retention and cessation success. However, minimal application of evidence-based behavior change frameworks, inconsistent reporting of engagement metrics, reliance on self-reported abstinence, and scalability limitations were noted.

**Conclusions:**

Digital interventions show promise for vaping cessation, particularly among youth and young adults, but current evidence highlights both opportunities and limitations. Effective interventions leverage personalization and social support to enhance engagement and quit outcomes. However, challenges such as high dropout rates, accessibility barriers, and limited use of rigorous evaluation methods persist. Future research should prioritize hybrid approaches that combine digital support with human interaction, apply equity-focused design principles, and adopt pragmatic, theory-driven evaluation methods to accelerate translation from pilot success to sustainable public health impact.

## Introduction

### Background

The rise in e-cigarette use (vaping), particularly among youth and young adults, has become a pressing public health issue worldwide. Frequently marketed as a tool for smoking cessation and a safer alternative to traditional tobacco products, vaping has become widely popular among nonsmokers and introduced a new generation to nicotine addiction [1-4]. The myriad of flavor options, convenient and discreet device designs, misconceptions about vaping-related harms, the addictive nature of nicotine, and complex socioenvironmental influences further perpetuate vaping behaviors and complicate cessation efforts [2-7]. This has prompted a call to action by the World Health Organization to prevent young people from vaping and promote cessation [8]. Despite the proliferation of vaping among youth and young adults, recent evidence indicates that many young e-cigarette users want to quit [9-13]. This expressed desire to quit, coupled with the challenges associated with vaping, necessitates the urgent development of tailored, accessible, and effective interventions to support individuals in their quit journeys.

Vaping cessation presents unique challenges that distinguish it from smoking cessation [13]. Barriers such as social influences, stress reduction, and sensory gratification overlap, yet key differences exist, including the appeal of flavored e-liquids, the discretion of vaping, and the lack of awareness of personal vaping habits. Many users also engage in dual use (vaping and smoking) or co-use with cannabis [14]. In addition, the lack of reliable information on vaping-related health risks fosters confusion and mistrust. These distinctions underscore the need for interventions tailored explicitly to the vaping context rather than relying solely on adaptations of smoking cessation tools [13].

Digital interventions have emerged as promising tools for behavior change, particularly for youth and young adults who are highly engaged with technology [15-22]. Mobile apps, text messaging, social media, and web-based platforms offer advantages such as real-time feedback, continuous support, and personalized content tailored to individual behavior, goals, and preferences. By leveraging technology, digital interventions may address key barriers to vaping cessation, such as social influences, by providing community support, peer-based encouragement, and real-time coaching [17-19]. While previous reviews have found that vaping cessation interventions that use digital technologies are successful in supporting quitting [15-18], including for youth [19], an in-depth exploration of these digital interventions remains lacking.

### Aim and Objective

This scoping review aimed to identify and map the landscape of digital interventions designed to support vaping cessation. By synthesizing existing research, this review sought to provide insights into the effectiveness, engagement strategies, and implementation considerations of digital vaping cessation interventions. This review aimed to inform future intervention development by highlighting best practices, gaps, and opportunities in the field. This scoping review addressed the following questions: (1) What types of digital interventions have been developed and/or evaluated for vaping cessation? (2) What evidence exists regarding the effectiveness of these digital interventions in promoting vaping cessation and user engagement? (3) What key barriers and facilitators influence the adoption, adherence, and success of digital vaping cessation interventions? (4) What gaps remain in the literature, and what areas should future research prioritize to enhance the design and effectiveness of digital vaping cessation tools? By addressing these questions, this review aimed to provide a comprehensive synthesis of digital vaping cessation tools, offering guidance for future research and intervention design to enhance accessibility, engagement, and long-term cessation success.

## Methods

### Study Design

Scoping reviews are well-suited to projects aiming to map out a broad and evolving field where the evidence is diverse and emerging [23]. Given that vaping cessation interventions are a relatively new area of research, this method allows for the inclusion of various study designs, including randomized controlled trials (RCTs), qualitative studies, protocols, and pilot programs [23]. This is crucial for understanding the effectiveness of these interventions and the challenges and opportunities they present. In addition, the rapid evolution of digital technology necessitates a broad and inclusive approach to ensure that the review captures the full range of available evidence. The Joanna Briggs Institute Reviewer’s Manual was used to guide the conduct of this scoping review [23]. The scoping review followed the PRISMA-ScR (Preferred Reporting Items for Systematic Reviews and Meta-Analyses extension for Scoping Reviews) checklist [24]. A scoping review protocol was drafted internally by KF and LS.

### Search Strategy

The lead author (KF) developed the search strategy in collaboration with an academic librarian to ensure comprehensive coverage of relevant literature. Preliminary searches were conducted in high-impact journals related to digital health and tobacco cessation to refine search terms. An initial set of searches in MEDLINE was performed to analyze title and abstract keywords and the index terms used to classify relevant papers. Based on these findings, a finalized list of search terms was compiled. A systematic search of the databases CINAHL, MEDLINE, and PsycINFO was conducted in October 2024 to identify peer-reviewed studies evaluating digital interventions for vaping cessation. Boolean operators (AND/OR) were used to refine search results, combining keywords related to vaping cessation and digital interventions, such as mobile health, text messaging, mobile apps, telehealth, and social media. The search strategy was structured to capture quantitative, qualitative, and mixed methods studies, ensuring a broad representation of intervention types and study designs. A hand search of reference lists from included studies was conducted to identify additional relevant papers. However, gray literature, conference abstracts, and non–peer-reviewed sources were not included, as this review focused on empirical evidence from peer-reviewed research to ensure methodological rigor. The full search strategy, including search terms and database queries, is provided in Multimedia Appendix 1.

### Eligibility Criteria

Included studies had to be primary research papers that provided evidence on digital interventions for vaping cessation. More specifically, studies had to examine mobile apps, text messaging programs, social media or web-based platforms, telehealth coaching, or other digital-based interventions explicitly designed to support individuals quitting vaping. To meet these criteria, studies had to report on at least one primary or secondary outcome related to vaping cessation, such as abstinence rates, reduction in vaping frequency, changes in nicotine dependence, user engagement, retention, adherence or intervention satisfaction. Studies that did not include user outcome data but systematically evaluated the design, quality, or behaviour change potential of vaping cessation tools were also included.

Studies that focused solely on smoking cessation were excluded unless they explicitly provided distinct data on vaping cessation. Likewise, studies that examined vaping prevention efforts, such as educational campaigns aimed at deterring initiation, were excluded, as this review focused on cessation rather than prevention. Research that only explored user preferences for digital vaping cessation interventions without evaluating an actual intervention was also excluded, as existing literature already addresses cessation method preferences [19-21]. In addition, studies that reported only on attitudes or knowledge about vaping without assessing behavioral change or specific interventions were excluded.

No restrictions were placed on participant age, allowing for the inclusion of interventions targeting adolescents, young adults, and older adults. Only peer-reviewed studies published in English after January 2018 were included to capture recent work in digital vaping cessation interventions. Studies that did not provide empirical data, such as editorials, opinion pieces, or gray literature, were excluded to maintain methodological rigor. This review aimed to provide a clear, actionable synthesis of tools and strategies developed and tested to support vaping cessation by focusing on existing digital interventions.

### Evidence Selection

Studies retrieved from the database searches were managed using Covidence (Veritas Health Innovation Ltd), a reference management and screening software. Any duplicates were removed by Covidence before being imported for screening. KF manually conducted a 2-stage screening process following duplicate removal. During level 1 screening, the titles and abstracts of the studies were assessed against the eligibility criteria. Studies that did not meet the criteria based on their title or abstract were excluded. In level 2 screening, the full-text papers that passed level 1 screening were reviewed to confirm their eligibility for inclusion. Studies that met all inclusion criteria were selected for full data extraction, while reasons for exclusion at the full-text stage were recorded, consistent with PRISMA-ScR guidelines [24]. For any studies where there was a question of whether the criteria were satisfied, KF consulted with LS, and a decision was made through discussion and consensus.

### Data Extraction

Data extraction was conducted manually by KF, following a standardized extraction form designed with LS to ensure consistency and accuracy. The extracted data were organized in Microsoft Word tables and managed using Zotero reference manager software. The key extracted variables included: (1) study characteristics (lead author, year of publication, country of origin, study design, sample size, and population demographics), (2) intervention details (type of digital intervention, duration, and delivery format), and (3) primary and secondary outcomes (vaping cessation rates, changes in nicotine dependence, intervention engagement, retention, and user satisfaction).

A second data extraction form was used to categorize intervention strategies and implementation considerations, focusing on (1) theoretical frameworks and behavior change models used in intervention design, (2) common features and engagement strategies within digital interventions, (3) barriers and facilitators to intervention success, and (4) implementation challenges and recommendations for future digital vaping cessation tools. The data extraction forms were developed iteratively, with modifications made after initial implementation on a sample of studies to refine data categorization and clarity.

### Analysis and Presentation of Results

A descriptive analysis of the included studies was conducted to address the objectives of this scoping review. A narrative synthesis was used to summarize key findings, categorize studies by intervention modality (eg, mobile apps, text messaging, telehealth, and social media), and evaluate their effectiveness, engagement strategies, and implementation considerations. Quantitative data, including cessation rates, engagement metrics, and user retention statistics, were summarized descriptively and tabulated to facilitate comparison. Qualitative data were analyzed using thematic synthesis, identifying recurring intervention strategies, barriers, and facilitators. Findings were presented using narrative descriptions, frequency distributions, and visual representations, where appropriate, to enhance clarity and comparability across intervention types.

### Ethical Considerations

This study was classified by the University of British Columbia Okanagan Behavioral Research Ethics Board as research not involving human participants and, therefore, not subject to institutional review board jurisdiction. All included studies were appropriately cited to respect intellectual property rights.

## Results

### Evidence Selection

**Figure 1.**
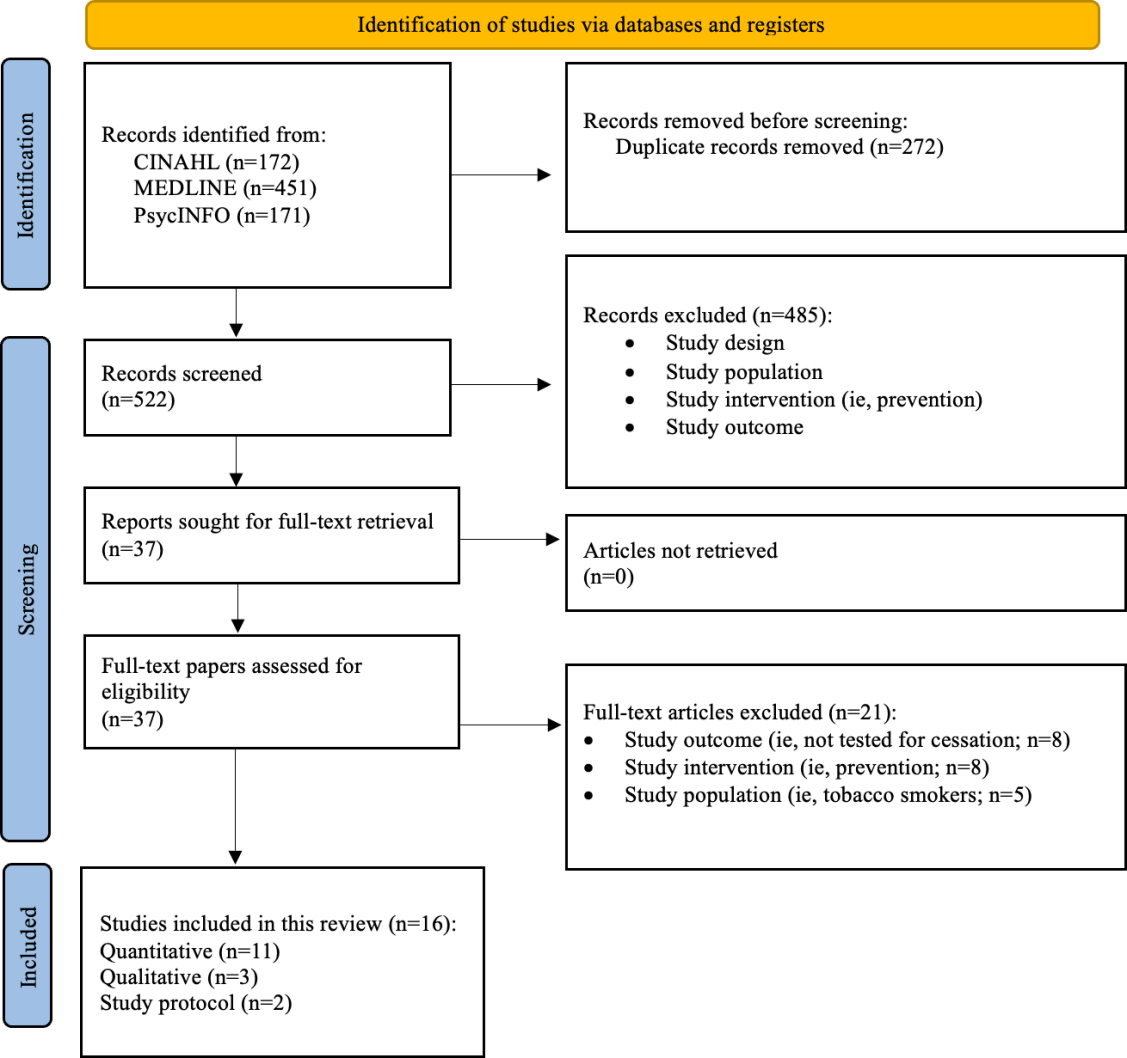
PRISMA (Preferred Reporting Items for Systematic Reviews and Meta-Analyses) flow diagram.

A total of 794 papers were identified through database searches (CINAHL: n=172, MEDLINE: n=451, and PsycINFO: n=171). After 272 duplicate records were removed, 522 papers remained for title and abstract screening. Of these, 485 studies were excluded for not meeting the eligibility criteria, leaving 37 full-text papers for further assessment. Following a full-text review, 21 studies were excluded due to irrelevant study outcomes (n=8), ineligible intervention types (n=8), or nonqualifying study populations (n=5). Ultimately, 16 studies met all inclusion criteria and were included in the final review. Figure 1 presents the PRISMA-ScR flow diagram, outlining the study selection process [24].

### Main Characteristics of the Included Papers

A total of 16 studies were included in this scoping review, published between 2018 and 2024, reflecting the timeframe of our search strategy rather than a true surge in research activity on digital vaping cessation interventions. The majority of studies were conducted in the United States (n=9), with additional studies from Canada (n=4) and Australia (n=1). This geographic distribution highlights a concentration of research in high-income countries, with limited representation from low- and middle-income regions. The target population of the digital vaping cessation interventions varied across studies. While most interventions focused on youth and young adults, a few studies included adults older than 30 years of age. Sample sizes ranged widely, from as few as 8 participants in pilot feasibility studies to over 2500 participants in large-scale trials. Participants were predominantly White, female, and younger than 30 years of age, emphasizing the need for future research to include more diverse populations to address health disparities in vaping cessation outcomes.

The primary outcome of interest across studies was vaping cessation, measured through self-reported abstinence and, in some cases, biochemical verification (Table 1). The table includes only the 9 studies that explicitly reported cessation outcomes; other studies are described narratively in the “Results” section. Secondary outcomes included reductions in vaping frequency, nicotine dependence, user engagement with the intervention, participant satisfaction, intervention retention or adherence, and intervention design. Studies varied in their use of behavior change theories, with some explicitly integrating cognitive behavioral therapy (CBT), the transtheoretical model, or social cognitive theory (SCT). In contrast, others lacked a clear theoretical foundation. The design and development of digital interventions often involve multidisciplinary teams, including researchers, clinicians, behavioral scientists, app developers, and public health experts. While some studies described user-centered design processes that engaged end users (eg, focus groups and co-design sessions), others did not specify how interventions were tailored to participant needs. The duration of intervention development and evaluation varied considerably, with some studies reporting development phases lasting under 6 months, while others described multiyear processes. Many studies did not specify the timeline for intervention development, limiting insights into how long it takes to design and refine practical digital vaping cessation tools. A detailed overview of the study characteristics, intervention types, and primary outcomes is provided in Multimedia Appendix 2.

**Table 1.** Summary of tested digital interventions and outcomes.

Study	Intervention type	Participants (n)	Mean age (years; SD)	Sex distribution, %	Outcomes
Bilic et al [25]	Zoom-based peer coaching	89	—^a^ (university students)	Female=73% Male=23.6% Other=3.4%	Reduced e-cigarette use (*b*=–2.1; *P*<.001) and lower use susceptibility (RR^b^=0.04; *P*=.05) compared with no coaching sessions.
Graham et al [26]	Text messaging program (“This is Quitting”)	1503	16.4 (0.8)	Female=50.6% Male=42.1% Other=7.4%	Participants receiving the intervention were significantly more likely to report 30-day abstinence at 7 months (37.8%) compared with the control group (28%).
Graham et al [27]	Text messaging program (“This is Quitting”)	2588	20.4 (1.7)	Female=50.3% Male=48.4% Other=1% Refused=0.2%	Participants receiving the intervention were significantly more likely to report 30-day abstinence at 7 months (24.1%) compared with the control group (18.6%).
Krishnan et al [28]	Text messaging program (“Quit the Vape”) ± counseling	58	27.3 (5.5)	Female=41.4% Male=53.5% Other=5.2%	The intervention with counselor-delivered messages reported higher rates of 7-day abstinence (27.8%) compared with those without a counselor group (11.1%) and control groups (5.9%).
Marler et al [29]	Mobile app (“Pivot”)	73	37.4 (11.3)	Female=52% Male=45% Other=3%	At 26 weeks, 45% of participants reported achieving 30-day vaping abstinence.
Orfin et al [30]	Text messaging program (“Kick Vaping”)	40	22.3 (2.1)	Female=45% Male=47.5% Other=7.5%	At month 3, 75% of participants reported 7-day vaping abstinence.
Palmer et al [31]	Telehealth ± contingency management (CM)	27	20.3 (1.2)	Female=66.7% Male=33.3% Other=0	Participants in the CM group submitted 55.4% abstinent samples compared with 8% in the control group, showing preliminary support for the effectiveness of the CM intervention. No difference was noted between the groups at follow-up.
Raiff et al [32]	Telehealth + CM	8	19.9 (1.5)	Female=63% Male=37% Other=0	All 8 participants quit vaping nicotine during the 2-week intervention, with 100% compliance in submitting samples and attending calls.
Webb et al [33]	Mobile App ("Quit Genius- Vaping") + nicotine replacement therapy (NRT)	51	27.9 (8.1)	Female=54.6% (percentages for male or other not provided)	At 1 month post-quit-date, 48.9% of participants reported 7-day abstinence, and 28.8% reported 30-day continuous abstinence.

a Not applicable.

b RR: relative risk.

### Types of Digital Interventions

Four categories of interventions were examined, including text messaging programs (n=4; 26-28,30], mobile apps (n=2; 29,33], telehealth-delivered contingency management (CM; n=2; 31,32], and web- or social media–based support (n=1; 25]. McKay et al [34] and Sanchez et al [35] conducted assessments and content analyses of vaping cessation mobile apps available on the Apple and Google Play stores. The 2 study protocols sought to test an Instagram (Meta)-based intervention [36] and a mobile app–based intervention, “Crush the Crave” [37]. In addition, Huma et al [22], Lyu et al [38], and Struik et al [39] qualitatively explored app-based, social media–based, and web-based cessation support, respectively.

#### Text Messaging Interventions

Text messaging programs emerged as a modality for vaping cessation, demonstrating high feasibility and efficacy across diverse populations. “This Is Quitting*”* [26,27], an automated, SMS text messaging program was designed for youth vaping cessation and tailored to users’ age, quit date, and vape brand. Grounded in SCT and youth smoking cessation research, it provides skill-building, coping strategies, mental health support, and reinforcement of social norms through peer-written messages. The program delivered tailored messages before and after a quit date, focusing on the risks of vaping and the benefits of quitting, with on-demand support via keywords. When targeted toward young adults aged 18‐24 years, the “This is Quitting” program achieved a 24.1% 30-day point prevalence abstinence (PPA) rate at 7 months, significantly outperforming the control group at 18.6% [27]. Comparably, when trialed for adolescents ages 13‐17 years, “This is Quitting” achieved 37.8% abstinence, emphasizing the role of tailored and age-specific content [26].

“Kick Vaping” [30], built upon “Decídetexto,” a smoking cessation text messaging intervention for Latinos, was particularly impactful for Latino young adults, combining English and Spanish messaging to improve accessibility. “Kick Vaping*”* contains 212 messages delivered across 4 phases: prequit, quit day, postquit intensive, and postquit maintenance, as well as bidirectional communication with research staff and automated responses triggered by keywords. “Kick Vaping” reported 75% self-reported 7-day abstinence at 3 months and significantly increased self-efficacy among participants. Hybrid intervention “Quit the Vape” [28] integrated automated text messaging with optional live counsellor-delivered messages and interactions. The intervention with counselor-delivered messages reported higher rates of 7-day abstinence (27.8%) compared to the group without a counselor (11.1%) and the control group (5.9%), indicating that combining automated and human elements may serve to create a more personalized cessation experience and enhance efficacy [28].

#### Mobile App–Based Interventions

Mobile apps emerged as a versatile platform, leveraging a combination of behavioral tools for vaping cessation. In their qualitative descriptive study, Huma et al [22] found that young adults aged 20‐29 years preferred mobile vaping cessation tools that are accessible, personalized, and engaging. Desired features included behavioral tracking, tailored quit plans, peer support, motivational messages, and evidence-based education. Emotional support and interactive elements, such as gamification and progress milestones, were also highlighted for their potential in sustaining engagement [22].

The “Pivot*”* app, adapted from a successful digital tobacco cessation program, is a 12-month app-based intervention designed for vaping cessation [29]. It integrates evidence-based methods such as motivational interviewing, CBT, self-determination theory, and acceptance and commitment therapy. Key features include educational content, progress tracking, a moderated online discussion community, and personalized quit coaching. At 6 months, “Pivot” achieved a 45% 30-day PPA rate and demonstrated significant reductions in vaping dependency among nonabstinent participants. Secondary outcomes included improvements in self-efficacy, motivation to quit, and reduced vaping frequency and intensity [29]. The “Quit Genius-Vaping” (Digital Therapeutics, Inc) app [33] is a comparable hybrid program that combines self-guided CBT content with digital coaching and nicotine replacement therapy to support vaping cessation. Notably it was the only intervention to directly integrate the use of nicotine replacement therapy. The app provides users with tailored quit plans, educational modules, and access to professional coaching. In the pilot study, researchers found that one month after the participants’ target quit date, the program achieved 48.9% 7-day abstinence and 28.8% 30-day abstinence. Secondary outcomes included reductions in vaping frequency and improvements in user-reported confidence to quit [33]. While both “Pivot” [29] and “Quit Genius-Vaping” [33] demonstrated promising results in achieving vaping cessation, their findings are limited by relatively small sample sizes, with 73 and 51 participants, respectively.

Sanchez et al’s [37] protocol describes one of the first RCTs to evaluate the effectiveness of an app-based intervention for supporting vaping cessation among youth and young adults. Sanchez et al [37] proposed testing a third vaping cessation app, “Crush the Crave,” among youth aged 16‐18 years and young adults aged 19‐29 years. An earlier version of the “Crush the Crave” app was used for smoking cessation. “Crush the Crave” offers features such as tracking vaping triggers, cravings, and habits through a geotagged diary while providing users with supportive messages, personalized visual progress, and access to evidence-based resources such as quitlines [37]. Additional features include push notifications highlighting money saved, health improvements, and milestones achieved, as well as tailored messages and digital awards to encourage users throughout their quitting process. This study will assess self-reported 30-day PPA at 3 months as the primary outcome [37]. Sanchez et al [37] plan to include the intention to quit within 6 months, puffs per session, daily vaping frequency, and total vape sessions in the past 30 days as secondary measures.

Recent reviews specific to mobile app-based vaping cessation interventions highlight moderate quality and limited evidence-based design. Sanchez et al [35] conducted one of the first quality assessments and content analyses of free vaping cessation apps and found only a small number available, many of which adapted smoking cessation frameworks with little tailoring to vaping behaviors. Common features included tracking and motivational tools, but few incorporated quit planning, relapse prevention, or direct links to quitlines. Similarly, McKay et al [34] reviewed 30 apps in Australia and found that while most were user-friendly and included some behavior change features, few demonstrated the inclusion of credible health information, goal-setting tools, or alignment with emerging clinical guidance. Both analyses were based on app content and functionality rather than user engagement or the primary outcome of cessation, rather, they offered an overview of the scope, content and quality measures of vaping cessation apps currently available in comercial app stores [34,35].

#### Telehealth-Delivered CM

This scoping review examined 2 telehealth-delivered CM interventions [31,32]. CM involves delivering an incentive, often financial, contingent on objective evidence of abstinence. Because CM relies on incentives, it is essential to verify abstinence frequently. In the examined studies, vaping abstinence was verified using saliva cotinine (nicotine metabolite) samples. Participants in Raiff et al [32] tried and evaluated 2 different saliva cotinine tests (NicAlert and iScreen) to verify abstinence, with financial rewards provided upon confirmation (up to US $140). Participants were given information about quitting vaping via referral to a government-supported website [40] to help support their cessation efforts, as the telehealth calls did not focus on cessation coaching but rather on the evaluation and witnessing of the cotinine tests. All 8 participants in Raiff et al [32] achieved full vaping abstinence during a 2-week trial period, with 100% compliance in submitting samples and attending calls. In addition, iScreen was consistently rated higher on all measured dimensions, including ease of use, compared to NicAlert.

Palmer et al [31] conducted a similar study using cotinine-verified (iScreen) vaping abstinence paired with CM (up to US $200). Participants were instructed to download the DynamiCare (DynamiCare Health Inc) app; every 2‐3 days during the treatment period, the app prompted participants to submit videos of their saliva cotinine test collection and results. To augment the CM intervention and provide skills-based resources and content during the vaping quit attempt, all participants were provided with information for “This is Quitting” [26,27,31]. Whether participants’ experiences with “This is Quitting*”* influenced the overall cessation results is unclear. Participants in the CM group submitted 55.4% abstinent samples compared with 8% in the control group, showing preliminary support for the CM intervention [31]. However, the authors noted no difference between the intervention and control groups at follow-up. Furthermore, both studies underscore that the CM approach often requires robust infrastructure and resources for implementation [31,32].

#### Web- and Social Media–Based Support

Struik et al [39] explored the strengths and limitations of the web-based cessation program “QuitNow” through qualitative interviews with 36 participants who vape, smoke, or dual-use. “QuitNow*”* provides personalized quit plans, sessions with trained quit coaches, a coach-moderated community forum, multiple support channels (telephone, SMS text messaging, and live chat), and resources for users and health care professionals. Participants appreciated its evidence-based information, credibility, and social support features, particularly forums and quit coaches. However, noted limitations included a text-heavy interface, insufficient youth-oriented and vaping-specific content, and a lack of personalization. Recommendations included improving the platform’s aesthetics, incorporating mobile-friendly features, tailoring content to younger users, and providing additional content for those seeking vaping cessation [39].

Bilic et al [25] expanded the scope of web-based interventions by integrating Zoom (Zoom Video Communications, Inc)-based peer health coaching with multibehavioral support targeting vaping cessation. This approach addressed co-occurring health behaviors, such as physical activity and stress management, within the same framework. Participants highlighted the benefits of real-time online interaction and the flexibility of remote delivery. Preliminary outcomes suggested promising reductions in e-cigarette use and improved self-efficacy compared with no coaching. However, the study emphasized the need for larger, controlled trials to confirm these findings.

Lyu et al [38] identified Instagram as a suitable platform for delivering peer-based vaping cessation interventions due to its group messaging, direct messaging, and multimedia sharing capabilities. Participants expressed comfort using their personal social media accounts for mentoring, provided clear boundaries were maintained. Building on this, Lyu et al [36] detailed a protocol for an Instagram-based intervention targeting young adults through private groups. The intervention plans to include up to 3 posts per weekday for 25 days, incorporating techniques such as self-liberation, stimulus control, and counterconditioning while encouraging quit dates and detailed plans. Groups are to be facilitated by a trained cessation counselor with additional support from a pediatrician, and participants will be educated on nicotine dependence, with encouragement to consider pharmacotherapy via health care providers [36].

## Discussion

### Principal Findings

This scoping review synthesized evidence from 16 studies evaluating digital vaping cessation interventions, highlighting their potential and ongoing challenges. The findings demonstrate that text messaging programs, mobile apps, telehealth-based CM, and web-based or social media interventions offer promising strategies for supporting individuals in quitting vaping. Programs such as “Kick Vaping” and “Quit Genius-Vaping” achieved significant abstinence rates, indicating the effectiveness of personalized, behavior-based digital interventions in modifying vaping behaviors [30,33]. These findings align with the broader research on smoking cessation strategies, suggesting that tailored digital interventions targeting substance use can enhance engagement, self-efficacy, and quit success [41,42]. However, it is important to note that the current state of evidence remains far from robust, and that the included studies are limited by short follow-ups, small sample sizes, and attrition. Engagement metrics were variably reported, with completion rates and app logins most frequently measured, while duration of use and content-specific interactions were seldom reported, limiting comparability across interventions.

Two recurring design features emerged as particularly influential: social support and personalization. Social support was a cornerstone of many vaping cessation tools, leveraging peer mentoring, community networks, quit-coaching, or professional support [25,28,29,33,38,39]. For example, Lyu et al [38] emphasized the value of peer mentoring for adolescents, highlighting that effective mentors were those who had successfully quit vaping, were relatable, and provided regular check-ins. Inclusive and safe spaces, such as LGBTQ+ (lesbian, gay, bisexual, transgender, queer/questioning, and others)-friendly groups, were considered essential, along with a preference for small-group formats or one-on-one support. Similarly, Struik et al [39] reported that the “QuitNow*”* platform’s coach-moderated community forums and access to trained quit coaches were highly valued by participants, particularly among those who vaped or dual-used nicotine products. Programs such as “Pivot” [29], “Quit the Vape*”* [28] and “Quit Genius - Vaping” [33] demonstrated that hybrid models combining scalable digital tools with human coaching can strengthen accountability and foster connection. These findings highlight that social support is not simply an “add-on” but may be a key driver of motivation, particularly when programs create inclusive, safe, and culturally responsive spaces.

Personalization was also central to user engagement and sustained use. Programs such as “This Is Quitting” [26,27] tailored text messages based on users’ age, quit readiness, and vaping habits, while Webb et al [33] integrated individualized feedback, skill-building, and quiz content for the individual. Culturally adaptive approaches, such as bilingual support in “Kick Vaping*,*” underscored how tailoring content to linguistic and cultural preferences can reduce barriers and increase engagement among underserved populations [30]. However, gaps remain; Sanchez et al [35] observed that few interventions addressed vaping-specific behaviors, such as device type or flavored pod use, pointing to missed opportunities for relevance. Future programs should extend personalization beyond demographic tailoring to account for vaping culture, context, and product-specific behaviors.

A prominent limitation across studies was the inconsistent application of behavior change theories. CBT informed several mobile apps, providing structured strategies to identify triggers, challenge unhelpful thoughts, and set progress milestones [29,33]. The transtheoretical model supported tailored interventions aligned with readiness to quit [36], while SCT was implicitly present in peer-driven features that leveraged observational learning and self-efficacy [26,27]. Despite these examples, many studies failed to explicitly justify their theoretical choices or clarify how frameworks informed program features. This lack of transparency weakens transferability. Future developers should map intervention components (eg, tailored messaging, gamification, or social support features) to theoretical constructs, ensuring theory drives design and evaluation meaningfully rather than serving as a post hoc label. Recent reviews by Sanchez et al [35] and McKay et al [34] provide a complementary perspective on mobile app-based vaping cessation interventions. Both studies systematically assessed the quality and content of commercially available apps and found that many were user-friendly but lacked evidence-based content and behaviour change features such as quit planning or relapse prevention, illustrating that much of the publicaly available apps are limited in terms of clinical rigor and theoretical grounding.

User engagement and retention were identified as persistent challenges. Several studies reported high dropout rates, particularly after the initial novelty of the intervention diminished, as well as loss to follow-up [26,28]. Longer programs tended to experience greater attrition, suggesting feasibility trade-offs between length and depth of support and sustainability of participation. This reflects a broader challenge within mobile health research, where static content and passive engagement strategies often fail to sustain motivation [43]. Addressing this challenge will require adaptive and interactive approaches, including integrating dynamic content, in-time feedback, and progressive tailoring that evolves with user needs. Importantly, few interventions incorporated iterative, user-centered design processes; this limited tailoring may have contributed to reduced relevance and scalability.

Gamification and interactivity were underused in the design of most interventions. Although previous literature suggests these features can enhance digital health interventions [19,44-46], few vaping cessation tools incorporate them, thus limiting insight into their effectiveness. Milkowski et al [19] underscore the promise of gamification and how it resonates with youth; they suggest the exploration of virtual reality games as a viable avenue for vaping cessation support. In line with their findings and recommendations, we suggest cautious integration of gamification with rigorous evaluation. Interventions should consider which specific gamified elements (eg, rewards, milestones, and social competition) align with user needs and feasibility in real-world settings. To enhance real-world implementation, gamification features should be tested in pragmatic pilot settings to evaluate sustainability and acceptability, particularly in resource-limited contexts.

Another concern was the reliance on self-reported vaping abstinence, which may lead to overestimation of quit rates due to response bias. While the 2 CM studies incorporated the biochemical validation method of saliva cotinine testing, the rest of the studies relied solely on self-reported data, raising concerns about the accuracy of cessation outcomes [31,32]. Future studies should incorporate cost-efficient biochemical validation to strengthen confidence in findings and ensure methodological rigor. From an implementation perspective, CM interventions demonstrated high short-term efficacy but raised significant questions of scalability. The logistical and financial demands of incentive distribution limit widespread adoption. Embedding CM features into hybrid models or pairing smaller incentive structures with evidence-based behavioral supports may improve feasibility.

In addition, the promise of digital interventions should be balanced against the pitfalls. Overreliance on digital tools may exacerbate inequities for those without stable internet access or private device ownership. In addition, privacy and data security concerns may disproportionately impact youth and deter uptake, especially when interventions are delivered via social media platforms. Finally, while digital programs can increase accessibility, they should not fully replace human or clinical support due to the complexities that youth and young adults face in their cessation journeys. Overpromising the effectiveness of digital-only approaches risks undermining credibility and losing the confidence of users; a balanced approach that integrates digital reach with clinical support should therefore be considered.

Taken together, these findings underscore the importance of developing digital cessation tools that are theoretically grounded, user-centered, and rigorously evaluated. Hybrid approaches integrating automated digital support with tailored human interaction appear especially promising, combining scalability with personalization. Future research should prioritize co-design with diverse youth and young adult populations, culturally and linguistically tailored content, equity-focused delivery strategies, and pragmatic long-term follow-up. Addressing these priorities will be critical for translating promising pilot results into sustainable, population-level public health impact.

### Strengths and Limitations

This scoping review has several strengths that contribute to its value in understanding the current landscape of digital interventions for vaping cessation. A major strength is the comprehensive and systematic approach taken to identify and synthesize evidence from diverse study designs, ranging from RCTs to qualitative evaluations. This breadth allowed us to capture both effectiveness outcomes (eg, cessation rates) and user perspectives (eg, satisfaction, barriers, and facilitators). By focusing on recent literature, this review reflects the rapidly evolving nature of vaping cessation and digital health technologies. Another strength is the breadth of intervention types reviewed. This diversity of intervention modalities, including text messaging programs, mobile apps, telehealth platforms, and social media or web-based interventions, further strengthens the comprehensiveness of the synthesis.

However, there are notable limitations. Screening and data extraction were conducted by a single reviewer, which may introduce selection bias and reduce methodological rigor. While consensus discussions with a senior researcher mitigated this somewhat, a dual independent review would have enhanced reliability. Another limitation is that, while there is overlap between MEDLINE, Scopus, and Web of Science, we did not use Scopus or Web of Science for our formal search, which may have resulted in missed studies. In addition, excluding gray literature and non–peer-reviewed studies may have limited the ability to capture some real-world and emerging interventions. Although this decision was intended to maintain rigor, it may have excluded practical insights from implementational contexts. Consistent with scoping review methodology, no formal quality appraisal of included studies was conducted, which means the robustness of the evidence base cannot be fully evaluated [23]. Future reviews would benefit from determining the quality of the evidence.

Furthermore, most studies relied on self-reported outcomes and short follow-up periods, limiting confidence in long-term effectiveness. Publication bias may also be present, as studies with null or negative results are less likely to appear in the peer-reviewed literature. Finally, many included studies were conducted with small, homogeneous samples, primarily White and younger than 30 years of age. This limits the generalizability and highlights the need for more diverse populations in future research. Several interventions required participants to indicate motivation or intent to quit vaping. Few explicitly engaged youth who are not yet motivated to quit, which represents a missed opportunity to reduce the burden. Taken together, these strengths and limitations underscore the need for rigorous, inclusive, and methodologically transparent research to advance the field of digital vaping cessation.

### Conclusion

Digital interventions offer a potential pathway to support vaping cessation, providing accessible and personalized tools that can meet youth where they are. This scoping review highlights encouraging findings from text messaging programs, mobile apps, telehealth-based CM, and web-based platforms; however, the overall evidence remains preliminary and methodologically limited. Attrition, inconsistent reporting of engagement metrics, reliance on self-reported outcomes, and scalability concerns (particularly for CM) underscore the need for cautious interpretation.

Moving forward, digital interventions should work toward moving beyond proof-of-concept trials to prioritize (1) hybrid approaches that integrate digital features with tailored human support; (2) transparent application of behavior change frameworks, with clear mapping of program theoretical constructs; (3) equity-focused co-design that incorporates the perspectives of diverse youth populations, including underserved and marginalized groups; and (4) pragmatic, long-term evaluations that combine biochemical verification with real-world implementation testing. By addressing these priorities, digital cessation interventions can evolve in a more rigorous and user-centered direction; ultimately, strengthening their capacity to support the health and well-being of individuals seeking to quit vaping.

## Supplementary material

10.2196/76983 Multimedia Appendix 1 Literature search strategy.

10.2196/76983 Multimedia Appendix 2 Data extraction table.

10.2196/76983 Checklist 1 PRISMA-ScR (Preferred Reporting Items for Systematic Reviews and Meta-Analyses extension for Scoping Reviews) checklist.

## Abbreviations

CBT: cognitive behavioral therapy
CM: contingency management
LGBTQ+: lesbian, gay, bisexual, transgender, queer/questioning, and others
PPA: point prevalence abstinence
PRISMA-ScR: Preferred Reporting Items for Systematic Reviews and Meta-Analyses extension for Scoping Reviews
RCT: randomized controlled trial
SCT: social cognitive theory

## References

[1] Askwith Z, Grignon J, Ismail M (2024). Environmental influences on e-cigarette use among young people: a systematic review. Health Place.

[2] Crane LA, Asdigian NL, Fitzgerald MD (2023). Looking cool, doing tricks, managing stress, and nicotine addiction: youth perspectives on nicotine vaping and implications for prevention. Am J Health Promot.

[3] Coen SE, Nelson Ferguson K, Burke SM (2023). Teens talk vaping: a co-produced participatory study exploring teens’ reflections on vaping experiences and exposures in their everyday environments. SSM Qual Res Health.

[4] Guerra Castillo C, Hoeft KS, Couch ET, Urata J, Halpern-Felsher B, Chaffee BW (2024). Adolescents’ experiences and perceptions of e-cigarettes and nicotine addiction. Subst Use Misuse.

[5] Han G, Son H (2022). A systematic review of socio-ecological factors influencing current e-cigarette use among adolescents and young adults. Addict Behav.

[6] McCausland K, Maycock B, Leaver T, Jancey J (2019). The messages presented in electronic cigarette-related social media promotions and discussion: scoping review. J Med Internet Res.

[7] Skinner AT, Golonka M, Godwin J, Kwiatek S, Sweitzer M, Hoyle RH (2024). My friends made me do it: peer influences and different types of vaping in adolescence. Addict Behav.

[8] (2022). Electronic cigarettes: call to action. World Health Organization.

[9] Amato MS, Bottcher MM, Cha S, Jacobs MA, Pearson JL, Graham AL (2021). “It’s really addictive and I’m trapped:” a qualitative analysis of the reasons for quitting vaping among treatment-seeking young people. Addict Behav.

[10] Cha S, Amato MS, Papandonatos GD (2024). Changes over time in reasons for quitting vaping among treatment-seeking young people from 2019 to 2022. Addict Behav Rep.

[11] Cuccia AF, Patel M, Amato MS, Stephens DK, Yoon SN, Vallone DM (2021). Quitting e-cigarettes: quit attempts and quit intentions among youth and young adults. Prev Med Rep.

[12] Lin C, Mathur Gaiha S, Halpern-Felsher B (2024). E-cigarette and combustible cigarette cessation patterns, reasons, and methods among adolescents, young adults, and adults. Addict Behav.

[13] Sanchez S, Kaufman P, Pelletier H (2021). Is vaping cessation like smoking cessation? A qualitative study exploring the responses of youth and young adults who vape e-cigarettes. Addict Behav.

[14] Berg CJ, Krishnan N, Graham AL, Abroms LC (2021). A synthesis of the literature to inform vaping cessation interventions for young adults. Addict Behav.

[15] Buela PJ, Leelavathi L, Arumugham I. M (2025). Digital and pharmacological interventions for vaping cessation in young adults: a systematic review. JPMS.

[16] Butler AR, Lindson N, Livingstone-Banks J (2025). Interventions for quitting vaping. Cochrane Database of Systematic Reviews.

[17] Heshmati J, Pandey A, Benjamen J (2025). Vaping cessation interventions: a systematic review and meta-analysis. Tob Control.

[18] Kundu A, Kouzoukas E, Zawertailo L (2023). Scoping review of guidance on cessation interventions for electronic cigarettes and dual electronic and combustible cigarettes use. cmajo.

[19] Milkowski M, Olesk J, Asfura DM (2025). Youth vaping in the digital age: a systematic review of technological interventions for prevention and cessation.

[20] Amin S, Pokhrel P, Elwir T, Mettias H, Kawamoto CT (2023). A systematic review of experimental and longitudinal studies on e-cigarette use cessation. Addict Behav.

[21] Dai HD, Hanh P, Guenzel N, Morgan M, Kerns E, Winickoff JP (2023). Adoption of Vaping Cessation Methods by US Adolescent E-Cigarette Users. Pediatrics.

[22] Huma ZE, Struik L, Bottorff JL, Hasan MK (2022). Preferences for mobile-supported e-cigarette cessation interventions among young adults: qualitative descriptive study. JMIR Form Res.

[23] Peters MDJ, Godfrey C, McInerney P, Munn Z, Tricco AC, Khalil H, Aromataris E, Munn Z (2020). JBI Manual for Evidence Synthesis.

[24] Tricco AC, Lillie E, Zarin W (2018). PRISMA extension for scoping reviews (PRISMA-ScR): checklist and explanation. Ann Intern Med.

[25] Bilic A, Burns RD, Bai Y, Brusseau TA, Lucero JE, King Jensen JL (2023). Preliminary efficacy of a multi-behavioral zoom-based peer health coaching intervention in young adults: a stepped wedge randomized controlled trial. Cyberpsychol Behav Soc Netw.

[26] Graham AL, Cha S, Jacobs MA (2024). A vaping cessation text message program for adolescent e-cigarette users: a randomized clinical trial. JAMA.

[27] Graham AL, Amato MS, Cha S, Jacobs MA, Bottcher MM, Papandonatos GD (2021). Effectiveness of a vaping cessation text message program among young adult e-cigarette users: a randomized clinical trial. JAMA Intern Med.

[28] Krishnan N, Berg C, Le D, Ahluwalia J, Graham A, Abroms L (2023). A pilot randomized controlled trial of automated and counselor-delivered text messages for e-cigarette cessation. Tob Prev Cessation.

[29] Marler JD, Fujii CA, Utley MT, Balbierz DJ, Galanko JA, Utley DS (2024). Outcomes of a comprehensive mobile vaping cessation program in adults who vape daily: cohort study. JMIR Form Res.

[30] Orfin RH, Ramos Santiago JW, Decena Soriano R (2024). Kick Vaping: feasibility, acceptability, and preliminary impact of a vaping cessation text messaging intervention for Latino young adults. J Ethn Subst Abuse.

[31] Palmer AM, Tomko RL, Squeglia LM (2022). A pilot feasibility study of a behavioral intervention for nicotine vaping cessation among young adults delivered via telehealth. Drug Alcohol Depend.

[32] Raiff BR, Newman ST, Upton CR, Burrows CA (2022). The feasibility, acceptability, and initial efficacy of a remotely delivered, financial-incentive intervention to initiate vaping abstinence in young adults. Exp Clin Psychopharmacol.

[33] Webb J, Lin YT, Ang A (2023). Feasibility and preliminary outcomes of a mobile intervention combining cognitive behavioral therapy, virtual coaching, and nicotine replacement therapy for nicotine vaping cessation. Telemed Rep.

[34] McKay F, Chan L, Cerio R (2024). Assessing the quality and behavior change potential of vaping cessation apps: systematic search and assessment. JMIR Mhealth Uhealth.

[35] Sanchez S, Kundu A, Limanto E, Selby P, Baskerville NB, Chaiton M (2022). Smartphone apps for vaping cessation: quality assessment and content analysis. JMIR Mhealth Uhealth.

[36] Lyu JC, Olson SS, Ramo DE, Ling PM (2022). Delivering vaping cessation interventions to adolescents and young adults on Instagram: protocol for a randomized controlled trial. BMC Public Health.

[37] Sanchez S, Deck A, Baskerville NB, Chaiton M (2023). Supporting youth vaping cessation with the crush the crave smartphone app: protocol for a randomized controlled trial. JMIR Res Protoc.

[38] Lyu JC, Afolabi A, White JS, Ling PM (2022). Perceptions and aspirations toward peer mentoring in social media-based electronic cigarette cessation interventions for adolescents and young adults: focus group study. JMIR Form Res.

[39] Struik L, Christianson K, Khan S, Sharma RH (2023). Strengths and limitations of web-based cessation support for individuals who smoke, dual use, or vape: qualitative interview study. JMIR Form Res.

[40] (2025). How to quit vaping. Smokefree Teen.

[41] Shams F, Wong JSH, Nikoo M (2021). Understanding eHealth cognitive behavioral therapy targeting substance use: realist review. J Med Internet Res.

[42] Whittaker R, McRobbie H, Bullen C (2019). Mobile phone text messaging and app-based interventions for smoking cessation. Cochrane Database of Systematic Reviews.

[43] Brindal E, Hendrie GA, Freyne J, Noakes M (2018). Incorporating a static versus supportive mobile phone app into a partial meal replacement program with face-to-face support: randomized controlled trial. JMIR Mhealth Uhealth.

[44] El-Hilly AA, Iqbal SS, Ahmed M (2016). Game on? Smoking cessation through the gamification of mHealth: a longitudinal qualitative study. JMIR Serious Games.

[45] Hadley W, Houck C, Brown LK, Spitalnick JS, Ferrer M, Barker D (2019). Moving beyond role-play: evaluating the use of virtual reality to teach emotion regulation for the prevention of adolescent risk behavior within a randomized pilot trial. J Pediatr Psychol.

[46] Xu L, Shi H, Shen M (2022). The effects of mHealth-based gamification interventions on participation in physical activity: systematic review. JMIR Mhealth Uhealth.

