# Supplementary material for: Exploration of Digital Interventions for Vaping Cessation: Scoping Review
Source: J Med Internet Res. 2025 Oct 23;27:e76983. doi: 10.2196/76983 (PMC12548964; doi:10.2196/76983)
Supplement: Multimedia Appendix 1 [file jmir-v27-e76983-s001.docx]

**Multimedia Appendix 2**

Literature Search Strategy

| **Database** | **Concepts** | **MeSH or Equivalent terms** | **Search Strategy** | **Number of Articles Identified** |
| --- | --- | --- | --- | --- |
| CINAHL EBSCO | Concept 1: vaping | (MH "Electronic Cigarettes") OR (MH "Vaping") | S1: (MH "Electronic Cigarettes") OR (MH "Vaping") | 4,037 |
|  |  | Keywords: vaping or vape or e-cigarette or electronic cigarette or electronic nicotine delivery system | S2: Vaping OR Vape OR E-cig* OR “Electronic nicotine delivery system*” | 6,914 |
|  | Concept 2: digital technology | (MH "Digital Technology+") OR (MH "Augmented Reality") OR (MH "Virtual Reality+") OR (MH "Virtual Reality Exposure Therapy") OR (MH "Telehealth+") OR (MH "Mobile Applications") OR (MH "Smartphone") OR (MH "Text Messaging+") OR (MH "Social Media+") | S3: (MH "Digital Technology+") OR (MH "Augmented Reality") OR (MH "Virtual Reality+") OR (MH "Virtual Reality Exposure Therapy") OR (MH "Telehealth+") OR (MH "Mobile Applications") OR (MH "Smartphone") OR (MH "Text Messaging+") OR (MH "Social Media+") | 95,147 |
|  |  | Keywords: text messaging, mobile applications, smartphone, augmented reality, virtual reality, immersive technologies, telehealth, e-health, digital interventions, social media | S4: (“Text messag*” OR "SMS" OR "short message service” OR “Mobile app*” OR “Smartphone app*” OR “Augmented reality” OR "AR" OR “Virtual reality” OR "VR" OR “E-health” OR "mHealth" OR "mobile health" OR "digital health" OR "Telehealth" OR "Telemedicine" OR "Social media" OR "Facebook" OR "Twitter" OR "Instagram" OR “Reddit” "YouTube" OR “Immersive tech*” OR “digital tech*” OR "Wearable tech*" OR "fitness tracker*" OR "smartwatch*") | 122,512 |
|  | Concept 3: cessation | Keywords: cessation, quit, quitting, stop, stopping, reduction, intervention | S5: (cessation OR quit* OR reduc* OR stop* OR intervention) | 1,255,347 |
|  |  | Vaping MH OR Keywords | S6: S1 OR S2 | 7,837 |
|  |  | Digital Technolgy+ MH OR Keywords | S7: S3 OR S4 | 134,429 |
|  |  | Vaping AND Digital Technology AND Cessation | S8: S5 AND S6 AND S7  Publication Date: 20180101-; English Language | 172 |
| MEDLINE EBSCO | Concept 1: vaping | (MH "Vaping") OR (MH "Electronic Nicotine Delivery Systems") | S1: (MH "Vaping") OR (MH "Electronic Nicotine Delivery Systems") | 9,788 |
|  |  | Keywords: vaping or vape or e-cigarette or electronic cigarette or electronic nicotine delivery system | S2: Vaping OR Vape OR E-cig* OR “Electronic nicotine delivery system*” | 13,544 |
|  | Concept 2: digital technology | (MH "Digital Technology") OR (MH "Digital Health") OR (MH "Augmented Reality") OR (MH "Virtual Reality+") OR (MH "Virtual Reality Exposure Therapy") OR (MH "Telemedicine+") OR (MH "Mobile Applications") OR (MH "Smartphone") OR (MH "Text Messaging") OR (MH "Social Media") | S3: (MH "Digital Technology") OR (MH "Digital Health") OR (MH "Augmented Reality") OR (MH "Virtual Reality+") OR (MH "Virtual Reality Exposure Therapy") OR (MH "Telemedicine+") OR (MH "Mobile Applications") OR (MH "Smartphone") OR (MH "Text Messaging") OR (MH "Social Media") | 97,933 |
|  |  | Keywords: text messaging, mobile applications, smartphone, augmented reality, virtual reality, immersive technologies, telehealth, e-health, digital interventions, social media | S4: (“Text messag*” OR "SMS" OR "short message service” OR “Mobile app*” OR “Smartphone app*” OR “Augmented reality” OR "AR" OR “Virtual reality” OR "VR" OR “E-health” OR "mHealth" OR "mobile health" OR "digital health" OR "Telehealth" OR "Telemedicine" OR "Social media" OR "Facebook" OR "Twitter" OR "Instagram" OR “Reddit” "YouTube" OR “Immersive tech*” OR “digital tech*” OR "Wearable tech*" OR "fitness tracker*" OR "smartwatch*") | 604,843 |
|  | Concept 3: cessation | Keywords: cessation, quit, quitting, stop, stopping, reduction, intervention | S5: (cessation OR quit* OR reduc* OR stop* OR intervention) | 6,054,960 |
|  |  | Vaping MH OR Keywords | S6: S1 OR S2 | 13,544 |
|  |  | Digital Technolgy+ MH OR Keywords | S7: S3 OR S4 | 614,836 |
|  |  | Vaping AND Digital Technology AND Cessation | S8: S5 AND S6 AND S7  Publication Date: 20180101-; English Language | 451 |
| PsycINFO EBSCO | Concept 1: vaping | DE "Vaping" OR DE "Electronic Cigarettes" | S1: DE "Vaping" OR DE "Electronic Cigarettes" | 3,712 |
|  |  | Keywords: vaping or vape or e-cigarette or electronic cigarette or electronic nicotine delivery system | S2: Vaping OR Vape OR E-cig* OR “Electronic nicotine delivery system*” | 4,441 |
|  | Concept 2: digital technology | DE "Digital Technology" OR DE "Electronic Health Services" OR DE "Mobile Health" OR DE "Mobile Health Applications" OR DE "Mobile Phones" OR DE "Mobile Technology" OR DE "Virtual Reality" OR DE "Virtual Reality Exposure Therapy" OR DE "Augmented Reality" OR DE "Social Media" OR DE "Text Messaging" OR DE "Telemedicine" | S3: DE "Digital Technology" OR DE "Electronic Health Services" OR DE "Mobile Health" OR DE "Mobile Health Applications" OR DE "Mobile Phones" OR DE "Mobile Technology" OR DE "Virtual Reality" OR DE "Virtual Reality Exposure Therapy" OR DE "Augmented Reality" OR DE "Social Media" OR DE "Text Messaging" OR DE "Telemedicine" | 57,722 |
|  |  | Keywords: text messaging, mobile applications, smartphone, augmented reality, virtual reality, immersive technologies, telehealth, e-health, digital interventions, social media | S4: (“Text messag*” OR "SMS" OR "short message service” OR “Mobile app*” OR “Smartphone app*” OR “Augmented reality” OR "AR" OR “Virtual reality” OR "VR" OR “E-health” OR "mHealth" OR "mobile health" OR "digital health" OR "Telehealth" OR "Telemedicine" OR "Social media" OR "Facebook" OR "Twitter" OR "Instagram" OR “Reddit” "YouTube" OR “Immersive tech*” OR “digital tech*” OR "Wearable tech*" OR "fitness tracker*" OR "smartwatch*") | 99,471 |
|  | Concept 3: cessation | Keywords: cessation, quit, quitting, stop, stopping, reduction, intervention | S5: (cessation OR quit* OR reduc* OR stop* OR intervention) | 1,079,725 |
|  |  | Vaping MH OR Keywords | S6: S1 OR S2 | 4,655 |
|  |  | Digital Technolgy+ MH OR Keywords | S7: S3 OR S4 | 103,569 |
|  |  | Vaping AND Digital Technology AND Cessation | S8: S5 AND S6 AND S7  Publication Date: 20180101-; English Language | 171 |

Inclusion and Exclusion Criteria

| **INCLUSION** | **EXCLUSION** |
| --- | --- |
| Studies focusing on individuals of all ages | Grey literature |
| Published after January 2018, to capture to rise of digital interventions and the shift in the vaping landscape | Articles not related to vaping or focused on traditional tobacco cigarette cessation |
| Language: English | Articles that only describe the development of technology or interventions without any testing or application for vaping cessation. |
| Empirical journal articles (qualitative, quantitative, and mixed-methods research) and reviews |  |
