# Supplementary material for: Exploration of Digital Interventions for Vaping Cessation: Scoping Review
Source: J Med Internet Res. 2025 Oct 23;27:e76983. doi: 10.2196/76983 (PMC12548964; doi:10.2196/76983)
Supplement: Multimedia Appendix 2 [file jmir-v27-e76983-s002.docx]

**Multimedia Appendix 3**

Data Extraction Table

| **Publication** | **Country** | **Aim/Purpose** | **Design and Data Collection** | **Sampling and Recruitment** | **Key Findings** | **Limitations** |
| --- | --- | --- | --- | --- | --- | --- |
| Bilic, A., Burns, R. D., Bai, Y., Brusseau, T. A., Lucero, J. E., & King Jensen, J. L. (2023). Preliminary efficacy of a multi-behavioral Zoom-based peer health coaching intervention in young adults: A stepped wedge randomized controlled trial. *Cyberpsychology, Behavior, and Social Networking, 26*(9), 698-705. https://doi.org/10.1089/cyber.2022.0365 | United States | To test the preliminary efficacy of a Zoom-based peer coaching intervention on health and risk behaviours in young adults. | Stepped Wedge Randomized Control Trial    Data was collected via self-reported surveys at baseline, after one coaching session, and after two sessions using standardized questionnaires | Convenience sample of students recruited from one U.S. university  (*N*=89) | After two 1-hour Zoom based peer coaching sessions using the motivational interviewing approach, participants exhibited reduced e-cigarette use (*b* = -2.1, p < 0.001) and lower use susceptibility (RR = 0.04, p = 0.05) compared with no coaching sessions. Participants also indicated enhanced use of stress reduction techniques and an increase in vigorous physical activity. | Self-reported data may introduce response bias, as all behaviors were measured via questionnaires.  Small sample size and lack of power analysis may limit the ability to detect all significant differences. Convenience sampling and inclusion of only one university limits generalizability.  Differences in the demographic composition between groups (e.g., sex, race) could potentially bias the results despite randomization efforts. |
| Graham, A. L., Cha, S., Jacobs, M. A., Amato, M. S., Funsten, A. L., Edwards, G., & Papandonatos, G. D. (2024). A vaping cessation text message program for adolescent e-cigarette users: A randomized clinical trial. *JAMA*, *332*(9), 713-721. doi:10.1001/jama.2024.11057 | United States | To compare to effectiveness of a text message program for nicotine vaping cessation among adolescents | Randomized Clinical Trial (RCT)- Double-blinded parallel, 2-group individually randomized.    Data was collected through self-reported surveys over 7 months. The primary outcome was self-reported 30-day vaping abstinence. | Convenience sampling of adolescents recruited via Snapchat, Facebook and Twitter ads.  (*N*= 1,503; mean age= 16.4 years) | At 7 months, 37.8% of participants in the intervention group reported 30-day vaping abstinence compared to 28.0% in the control group. Repeated abstinence rates were significantly higher in the intervention group (17.3%) compared to the control group (8.2%). The intervention demonstrated effectiveness across different levels of nicotine dependence, mental health, and psychosocial adversity, making it a promising, scalable method for promoting vaping cessation among adolescents. | Self-reported abstinence was not biochemically verified, potentially leading to reporting bias. The intervention group may have been subject to social desirability bias due to more program touchpoints. The study's findings are generalizable only to adolescents interested in quitting, limiting applicability to other groups. Additionally, analyses of mediators and changes in cannabis and alcohol use were not included, which could provide further insights into behavior changes. |
| Graham, A. L., Amato, M. S., Cha, S., Jacobs, M. A., Bottcher, M. M., & Papandonatos, G. D. (2021). Effectiveness of a vaping cessation text message program among young adult e-cigarette users: A randomized clinical trial. *JAMA Internal Medicine*, *181*(7), 923-930. doi:10.1001/jamainternmed.2021.1793 | United States | To determine the effectiveness of a text-message program for e-cigarette cessation among young adults (YAs). | Randomized Clinical Trial (RCT)- Double-blinded parallel, 2-group individually randomized.    Data was collected at baseline and after 7 months via self-reported surveys. The primary outcome was self-reported 30-day vaping abstinence. | Convenience sampling of YAs recruited via Facebook and Twitter ads.  (*N*= 2,588; mean age = 20.4 years) | Participants receiving the intervention were significantly more likely to report 30-day abstinence at 7 months (24.1%) compared to the control group (18.6%).  The intervention was effective across demographic variables, including nicotine dependence. These findings establish a benchmark for vaping cessation interventions, demonstrating that text messaging can be a scalable and cost-efficient tool for promoting vaping cessation. | No biochemical verification of abstinence, rather self-reported data, which may introduce bias.  The study did not include teenagers, the group with the highest rates of e-cigarette use, limiting the generalizability of findings to younger age groups.  The use of incentives for participation may have introduced some level of assessment reactivity, potentially influencing the outcomes. |
| Huma, Z. E., Struik, L., Bottorff, J. L., & Hasan, M. K. (2022). Preferences for mobile-supported e-cigarette cessation interventions among young adults: Qualitative descriptive study. *JMIR Formative Research*, *6*(4), e33640. <https://doi.org/10.2196/33640> | Canada | To develop an understanding of young adults’ (YAs) preferences and perceptions on app-based e-cigarette cessation interventions. | Qualitative descriptive approach.    Data was collected via semi-structured interviews | Purposive sampling of current YA e-cigarette users looking to quit via Kijiji, Castanet, and university web posting.  (*N*= 12; mean age= 25.2 years) | YAs value mobile vaping cessation tools with features like personalized quit plans, behavioral tracking, peer support, motivational messaging, and evidence-based education. Emotional support and gamification were key to maintaining engagement, while concerns about data privacy and usability highlighted the need for user-friendly, inclusive designs. Participants emphasized the importance of tools that are accessible, culturally sensitive, and adaptable to diverse needs to enhance effectiveness and long-term success. | Small sample size with limited cultural and socioeconomic diversity, reducing the applicability of findings to broader populations with varied needs and experiences. |
| Krishnan, N., Berg, C. J., Le, D., Ahluwalia, J., Graham, A. L., & Abroms, L. C. (2023). A pilot randomized controlled trial of automated and counselor-delivered text messages for e-cigarette cessation. *Tobacco prevention & cessation*, *9*. Doi: 10.18332/tpc/157598 | United States | To examine the acceptability and preliminary efficacy of the Quit the Vape (QTV), an automated text messaging program for vaping cessation, delivered with and without live text counseling. | Pilot Randomized Control Trial (RCT)    Data was collected via self-reported surveys at 4-weeks, measuring vaping cessation, quit attempts, and satisfaction with the program. | Stratified random sampling from a pre-existing cohort of participants recruited via social media from 6 metropolitan areas in the U.S. (*N* = 58; mean age = 27.3 years) | The QTV with counselor-delivered messages (QTV-C) group reported higher rates of 7-day abstinence (27.8%) compared to the QTV (11.1%) and control groups (5.9%). Quit attempts and program satisfaction were also higher in the QTV-C group. Both intervention groups reported high engagement and satisfaction, suggesting that live text counseling enhances the effectiveness of automated cessation programs. | The study was not statistically powered to detect significant differences between groups, limiting its generalizability. Self-reported data without biochemical verification may introduce bias. Participants were not required to be motivated to quit, which may affect the intervention's overall efficacy in a more targeted population. The short 4-week follow-up period also limited the assessment of long-term cessation outcomes. |
| Lyu, J. C., Afolabi, A., White, J. S., & Ling, P. M. (2022a). Perceptions and aspirations toward peer mentoring in social media–based electronic cigarette cessation interventions for adolescents and young adults: Focus group study. *JMIR Formative Research*, *6*(12), e42538. <https://doi.org/10.2196/42538> | United States | To understand from mentee and mentor perspectives the needs, expectations, and concerns of adolescents and young adults (AYAs) regarding peer mentoring to inform the development of social media–based peer mentoring interventions for e-cigarette cessation. | Qualitative    Data was collected via 7 focus groups sessions including four mentee groups and three potential mentor groups. | Purposive sampling with participants recruited from the "Quit the Hit" (QTH) program, an Instagram-based e-cigarette cessation intervention active in California, South Carolina, and Minnesota.  (*N= 26*; mean age = 19.4 years) | AYAs valued peer mentoring in social media-based vaping cessation programs, preferring mentors of similar age who had successfully quit vaping. Participants favored small-group and one-on-one mentoring on platforms like Instagram. Key mentor traits included emotional intelligence, communication skills, and inclusivity. Challenges included setting boundaries and maintaining professionalism, while incentives like certificates and monetary compensation were motivating. Peer mentoring was seen as a promising strategy to enhance engagement and retention in digital interventions. | Limitations include a small, demographically narrow sample recruited from the "Quit the Hit" program, which may not generalize to broader populations. Participants’ familiarity with Instagram potentially biased platform preferences. The self-selection of motivated individuals interested in mentoring limited insights from less engaged groups. Additionally, the study focused on perceptions rather than real-world implementation or efficacy of peer mentoring. |
| Lyu, J. C., Olson, S. S., Ramo, D. E., & Ling, P. M. (2022b). Delivering vaping cessation interventions to adolescents and young adults on Instagram: Protocol for a randomized controlled trial. *BMC Public Health*, *22*(1), 2311. <https://doi.org/10.1186/s12889-022-14606-7> | United States | To describe the protocol for a randomized controlled trial (RCT) testing the efficacy of an Instagram-based vaping cessation intervention for adolescents and young adults. | Study protocol for an RCT | Will utilize purposive sampling leveraging social media (Facebook) and community outreach    Estimated need of approximately N=500 participants based on power analysis and estimation of attrition rates | The intervention involves participants receiving a structured, Instagram-based vaping cessation program. This includes tailored, interactive content such as motivational posts, educational materials, and prompts encouraging engagement with the program. Peer mentors also provide support through direct messages and group discussions. Participants in the control condition will be directed to kickitca.org, a website offering links to chatline and texting cessation services. 7-day point prevalence abstinence will be assessed immediately, 3 months, and 6 months after the treatment. | Reliance on self-reported outcomes could impact accuracy. Additionally, focusing solely on Instagram may limit generalizability to youth and young adults who use other platforms or have less social media access. The lack of long-term follow-up may restrict insights into sustained vaping cessation outcomes. |
| Marler, J. D., Fujii, C. A., Utley, M. T., Balbierz, D. J., Galanko, J. A., & Utley, D. S. (2024). Outcomes of a comprehensive mobile vaping cessation program in adults Who vape daily: Cohort study. *JMIR formative research*. doi: 10.2196/57376 | United States | To evaluate the Pivot mobile vaping cessation program among adult daily vapers, assessing participant engagement, retention, changes in vaping attitudes and behavior, and participant feedback. | Open-label, single-arm cohort study conducted remotely.    Participants engaged with the Pivot app over 26 weeks. Data was self-reported via app and web-based questionnaires at baseline, every two weeks for 12 weeks, and at the 26-week follow-up. | Non-proportional quota sampling with recruitment through web media (Facebook, Google Ads, Reddit, Craigslist)  (*N* = 73; mean age = 37.4 years) | The study found that 45% of participants achieved 30-day vaping abstinence at 26 weeks, and 30% maintained continuous abstinence from week 12 through week 26. Participants reported increased confidence to quit and reduced dependence on e-cigarettes. Most participants (88%) made at least one quit attempt, and engagement with the app remained high, particularly with messaging the coach and using the online community. | The study lacked a control group, limiting conclusions about the effectiveness of the intervention. Recruitment challenges resulted in under-representation of certain demographics, including people who had never smoked and non-White participants. Additionally, reliance on self-reported data and compensating participants may have introduced bias. The findings are preliminary, and further research is needed to establish effectiveness. |
| McKay, F., Chan, L., Cerio, R., Rickards, S., Hastings, P., Reakes, K., O’Brien, T., & Dunn, M. (2024). Assessing the quality and behavior change potential of vaping cessation apps: Systematic search and assessment. *JMIR mHealth and uHealth, 12,* e55177–e55177. <https://doi.org/10.2196/55177> | Australia | To evaluate the quality, usability, and behavior change potential of smartphone apps designed to support vaping cessation. | Systematic search and assessment (content analysis) | 6 vaping cessation apps available on the Australian Apple iTunes and Google Play stores were assessed. | Most vaping cessation apps scored poorly on quality and behavior change potential, with limited adherence to evidence-based guidelines. While some apps included features like goal-setting and progress tracking, these were inconsistently applied. User engagement tools, such as gamification and peer support, showed potential but were underutilized. Significant gaps in app design and content were identified, highlighting the need for improved integration of evidence-based strategies and collaboration with healthcare professionals. | The study relied on app store descriptions, which may not reflect actual app functionality. The study did not assess user experiences or long-term effectiveness of the apps, limiting real-world applicability. Additionally, the evaluation excluded apps not available in English, potentially overlooking region-specific tools. The rapidly changing nature of app content also poses challenges in maintaining relevance and accuracy of the findings. |
| Orfin, R. H., Ramos Santiago, J. W., Decena Soriano, R., Romero Acosta, E., Bermudez, D., Rodriguez, Y. L., ... & Cartujano-Barrera, F. (2024). Kick Vaping: Feasibility, acceptability, and preliminary impact of a vaping cessation text messaging intervention for Latino young adults. *Journal of Ethnicity in Substance Abuse*, 1-13. https://doi.org/10.1080/15332640.2024.2397422 | United States (including Puerto Rico) | To assess the feasibility, acceptability and preliminary impact of *Kick Vaping* among Latino young adults. | Single-arm pilot study.    Participants received 212 text messages in four phases over three months. Data was collected via self-reported surveys at baseline and at Month 3 to evaluate vaping cessation, self-efficacy, and satisfaction with the program. | Convenience sampling via community outreach (e.g., malls, community-based organizations), radio interviews, and social media.  (*N* = 40; mean age = 22.3 years) | At month 3, 75% of participants reported 7-day vaping abstinence. Self-efficacy scores significantly improved from 30.65 at baseline to 50.11 at follow-up. Additionally, 88.9% of participants reported high satisfaction with the program. The intervention was found to be feasible and acceptable, showing promise in helping young Latinos quit vaping. | The study lacked a control group, relied on self-reported data without biochemical verification, and had a small sample size. These factors limit the generalizability of the findings. The study was also limited to young adults, so the results may not apply to other age groups. Social desirability bias may have influenced participants’ responses. |
| Palmer, A. M., Tomko, R. L., Squeglia, L. M., Gray, K. M., Carpenter, M. J., Smith, T. T., ... & McClure, E. A. (2022). A pilot feasibility study of a behavioral intervention for nicotine vaping cessation among young adults delivered via telehealth. *Drug and alcohol dependence*, *232*(109311), 1-7. https://doi.org/10.1016/j.drugalcdep.2022.109311 | United States | To conduct a feasibility and acceptability trail of contingency management (CM) delivered via telehealth for youth and young adult (YYA) nicotine vaping cessation. | Pilot feasibility trial using a randomized controlled trail design.    The intervention was delivered remotely via the DynamiCare Health app over 28 days, with financial incentives provided based on abstinence, verified through saliva cotinine tests. Data was collected through app-based submissions of saliva samples and self-reported quit attempts, along with usability ratings and follow-up assessments at one month. | Convenience sampling of YYA via Facebook, Instagram, and Craigslist.  (*N* = 27; mean age = 20.3 years) | The study found high feasibility and acceptability, with 93% of participants completing the 28-day intervention and 89% completing the 1-month follow-up. Participants in the CM group submitted 55.4% abstinent samples compared to 8% in the control group, showing preliminary support for the CM intervention. Usability ratings for the app were favorable, particularly among the CM group. While abstinence rates were not significantly different between groups at the end of treatment, the intervention showed potential for promoting short-term vaping cessation. | The study was limited by a small sample size and imbalanced randomization (22 CM, 5 Monitoring). The study was underpowered to detect significant differences in long-term cessation outcomes. Additionally, the resource-heavy nature of contingency management presents scalability challenges. |
| Raiff, B. R., Newman, S. T., Upton, C. R., & Burrows, C. A. (2022). The feasibility, acceptability, and initial efficacy of a remotely delivered, financial-incentive intervention to initiate vaping abstinence in young adults. *Experimental and clinical psychopharmacology*, *30*(5), 632-641. https://doi.org/10.1037/pha0000468 | United States | To evaluate the feasibility, acceptability, and preliminary efficacy of a remotely delivered contingency management (CM) intervention for vaping abstinence among young adults and to compare NicAlert and iScreen for remote verification. | Pilot study using single-case multiple-baseline design.    Participants provided saliva samples during video calls to verify vaping abstinence using NicAlert and iScreen cotinine tests. Financial incentives were provided based on negative cotinine results. Data was collected over a 2-weeks, with participants completing follow-up questionnaires. | Convenience sampling with recruitment done via university administered email and word of mouth.  (*N* = 8, mean age =19.88 years) | All eight participants quit vaping nicotine during the 2-week intervention, with 100% compliance in submitting samples and attending calls. iScreen cotinine tests were rated more favorably by participants compared to NicAlert in terms of ease of use and accuracy. The intervention was rated as highly acceptable, with participants expressing satisfaction with the financial incentives and video support. The study demonstrated the feasibility of remotely delivered, incentive-based vaping cessation programs. | The study had a small sample size and lacked a control group, which limits the generalizability and ability to draw strong conclusions about the intervention's efficacy. The study also relied on self-reported data and short-term outcomes (2 weeks), without long-term follow-up to assess sustained abstinence. Additionally, potential demand characteristics may have influenced outcomes, as social support was provided through video sessions. |
| Sanchez, S., Deck, A., Baskerville, N. B., & Chaiton, M. (2023). Supporting youth vaping cessation with the Crush the Crave smartphone app: Protocol for a randomized controlled trial. *JMIR Research Protocols, 12*(1), e42956. doi: 10.2196/42956 | Canada | To determine the effectiveness of the *Crush the Crave* app in supporting vaping cessation in comparison with an assessment-only control group. | Study protocol for a 2-arm, single-blind, parallel randomized control trial (RCT).    Qualitative interviews (n=25) will be conducted with participants in the intervention arm to gain insight on the usability and acceptability of the app. | Will utilize purposive sampling to recruit participants via the Vaping Dependence Cohort—an existing panel of youth enrolled in a prospective cohort study at the University of Toronto. Study invitations will be sent to 600 youth and young adult e-cigarette users and randomized between an intervention arm, which will be using *Crush the Crave* (n=300), and an assessment-only control arm (n=300) in a 1:1 ratio. | The primary outcome variable will be self-reported 30-day point prevalence abstinence at 3 months, operationalized as not having vaped, even a puff, in the last 30 days. The secondary measures will include the intention to quit smoking in the next 6 months (yes or no), number of puffs per vaping session, number of vape sessions per day, and number of sessions in the past 30 days. | The study was designed to include an assessment-only control group. As the app is publicly available, there is the risk of cross-contamination. Cross-contamination measures will be put in place to assess awareness of and engagement with the *Crush the Crave* app among control group participants. Focuses on nicotine vaping, findings may not be generalizable to cannabis vaping which is on the rise. |
| Sanchez, S., Kundu, A., Limanto, E., Selby, P., Baskerville, N. B., & Chaiton, M. (2022). Smartphone apps for vaping cessation: Quality assessment and content analysis. *JMIR mHealth and uHealth, 10*(3), e31309. <https://doi.org/10.2196/31309> | Canada | To examine the quality of free vaping cessation apps, their contents and features, popularity among users, and adherence to evidence-based principles. | Systematic search and assessment (content analysis) | 8 apps available on the Canadian Apple and Google Play stores were included: 3 were developed specifically for vaping cessation and 5 focused on smoking cessation while also claiming to address vaping cessation. | Most apps scored low in quality, particularly in engagement, functionality, and adherence to evidence-based guidelines. Critical features like coping mechanisms for withdrawal and relapse prevention were often absent, and behavior change techniques, such as goal-setting and motivational messaging, were inconsistently applied. While apps with gamification and social support features showed higher engagement potential, these were rarely implemented. The study emphasized the need for evidence-based, user-focused app design to improve effectiveness. | The study relied on publicly available app store data, which may not fully represent app functionality or content. It did not assess user experiences or long-term app effectiveness, limiting real-world applicability. The analysis excluded non-English apps, potentially missing culturally relevant tools. Additionally, the rapidly evolving app market may render findings outdated, highlighting challenges in maintaining the study's relevance over time. |
| Struik, L., Christianson, K., Khan, S., & Sharma, R. H. (2023). Strengths and limitations of web-based cessation support for individuals who smoke, dual use, or vape: Qualitative interview study. *JMIR Formative Research*, *7*, e43096. doi:10.2196/43096 | Canada | To understand the strengths and limitations of web-based cessation support offered through QuitNow. | Qualitative descriptive design.    Data was collected via semi-structured interviews and analyzed with conventional content analysis. | Convenience sampling with recruitment via Facebook and Instagram.  (*N* = 36, aged 17-58 years) | Participants identified several strengths of QuitNow, such as the breadth of information, nonjudgmental atmosphere, and professional appearance of the website. They valued the social support and quit coach access features. However, participants also noted limitations, including too much text, the need to create an account, and a lack of youth-specific content. Individuals who vape requested more information on vaping-specific health risks and quit strategies, while participants who dual-use or smoke desired more personalized support. | The study's limitations include a lack of gender diversity, as the sample only included men and women, with no nonbinary or gender-nonconforming participants. Additionally, most participants were non-QuitNow users, limiting their familiarity with the program. The study was limited to participants from British Columbia, which may affect the generalizability of the findings to other regions. A reliance on self-reported data and limited time spent reviewing the website may have influenced participants’ perceptions of the program. |
| Webb, J., Lin, Y. T., Ang, A., Michero, D., Majeed, A., Eisingerich, A., & Glasner, S. (2023). Feasibility and preliminary outcomes of a mobile intervention combining cognitive behavioral therapy, virtual coaching, and nicotine replacement therapy for nicotine vaping cessation. *Telemedicine Reports*, *4*(1), 48-52. https://doi.org/10.1089/tmr.2023.0009 | United States | To examine the feasibility and preliminary outcomes of an mHealth vaping cessation intervention. | Single arm pilot design.    The intervention was delivered via the Quit Genius-Vaping (QG-V) app over a 6-week period. Data were collected through online questionnaires at baseline and 1 month post-quit-date, measuring self-reported 7-day and 30-day abstinence, nicotine dependence, and user satisfaction. | Convenience sampling via Facebook.  (*N* = 51, mean age = 27.9 years) | At 1 month post-quit-date, 48.9% of participants reported 7-day abstinence, and 28.8% reported 30-day continuous abstinence. There was a significant reduction in vaping frequency, duration, and nicotine dependence. Participants highly rated the intervention's helpfulness, with 87% reporting that the QG-V app met their needs. The intervention was considered feasible and effective for supporting vaping cessation. | The study lacked a control group, limiting the ability to assess the intervention's effectiveness compared to other cessation methods. Self-reported data without biochemical verification may introduce bias. The short 1-month follow-up does not provide insights into long-term cessation outcomes. Additionally, the study's small sample size restricts the generalizability of findings. |
